# Effects of Lumbosacral Spinal Cord Epidural Stimulation for Standing after Chronic Complete Paralysis in Humans

**DOI:** 10.1371/journal.pone.0133998

**Published:** 2015-07-24

**Authors:** Enrico Rejc, Claudia Angeli, Susan Harkema

**Affiliations:** 1 Kentucky Spinal Cord Injury Research Center, University of Louisville, Louisville, Kentucky, United States of America; 2 Frazier Rehab Institute, Kentucky One Health, Louisville, Kentucky, United States of America; Hertie Institute for Clinical Brain Research, University of Tuebingen., GERMANY

## Abstract

Sensory and motor complete spinal cord injury (SCI) has been considered functionally complete resulting in permanent paralysis with no recovery of voluntary movement, standing or walking. Previous findings demonstrated that lumbosacral spinal cord epidural stimulation can activate the spinal neural networks in one individual with motor complete, but sensory incomplete SCI, who achieved full body weight-bearing standing with independent knee extension, minimal self-assistance for balance and minimal external assistance for facilitating hip extension. In this study, we showed that two clinically sensory and motor complete participants were able to stand over-ground bearing full body-weight without any external assistance, using their hands to assist balance. The two clinically motor complete, but sensory incomplete participants also used minimal external assistance for hip extension. Standing with the least amount of assistance was achieved with individual-specific stimulation parameters, which promoted overall continuous EMG patterns in the lower limbs’ muscles. Stimulation parameters optimized for one individual resulted in poor standing and additional need of external assistance for hip and knee extension in the other participants. During sitting, little or negligible EMG activity of lower limb muscles was induced by epidural stimulation, showing that the weight-bearing related sensory information was needed to generate sufficient EMG patterns to effectively support full weight-bearing standing. In general, electrode configurations with cathodes selected in the caudal region of the array at relatively higher frequencies (25–60 Hz) resulted in the more effective EMG patterns for standing. These results show that human spinal circuitry can generate motor patterns effective for standing in the absence of functional supraspinal connections; however the appropriate selection of stimulation parameters is critical.

## Introduction

Severe spinal cord injury (SCI) is associated with disability and a drastic decrease in quality of life for affected individuals [1]. Also, the economic impact of SCI is striking, estimated in billions of dollars annually only in the Unites States [2], with the greatest costs to those with the most severe injuries. SCI graded A or B by the International Standards for Neurological Classification of Spinal Cord Injury [3] are characterized as functionally motor complete because these individuals are unable to stand, walk or move their legs voluntarily. This diagnosis normally implies severe limitations for neurological and functional recovery [2,4]. For decades, efforts to improve this condition have focused on the mammalian spinal cord, which is able to generate locomotor output in absence of input from the brain by central pattern generation [5–7]. To promote this capability, epidural stimulation of the lumbosacral spinal cord after complete thoracic spinal cord transection has been successfully applied to facilitate standing [8–10] and stepping [11–15] in mammals. These studies demonstrated that, in the absence of supraspinal input, epidural stimulation of the lumbosacral spinal cord can promote effective locomotion pattern, and that sensory afferent input is crucial to control it.

Complete spinal rats were also able to achieve weight-bearing standing when the spinal cord was stimulated with specific frequencies and sets of electrodes using a high-resolution array implanted over spinal cord segments L2-S2 [8]. Rostral bipolar stimulation at lower frequencies (10–15 Hz) produced vibratory movements but did not facilitate standing. Frequencies between 40 and 60 Hz resulted in the activation of extensor muscles, leading to partial weight-bearing standing. Stimulation at higher frequencies (80–100 Hz) resulted in non-specific movements with poor inter-limb coordination. On the other hand, bipolar caudal stimulation failed to facilitate weight-bearing standing at any frequency. These data highlighted the importance in providing specific stimulation parameters as well as weight-bearing related sensory information to the lumbosacral spinal cord in order to promote effective standing after SCI.

In humans, Dimitrijevic and colleagues showed that epidural stimulation of lumbosacral spinal cord can elicit tonic and rhythmic motor patterns of the lower limbs after motor complete SCI while lying supine [16–19]. Specifically, stimulation frequencies ranging from 5 to 15 Hz were found effective to initiate and retain lower-limb extension EMG patterns in individuals with motor complete SCI without any sensory information related to weight-bearing. Conversely, frequencies between 30 and 70 Hz were optimal to induce locomotor-like EMG activity in complete SCI individuals who lay supine or stepped using body weight support. Lumbosacral epidural stimulation, combined with the sensory input associated with weight-bearing, enabled a motor complete but sensory incomplete individual to progressively regain full weight-bearing standing with independence of leg extension, minimal self-assistance for balance and minimal external assistance for facilitating hip extension [20]. Stimulation effective for standing was focused on the caudal portion of the electrode array, using two different electrode configurations and stimulation frequencies equal to 15 and 25 Hz.

One aim of this study was to understand whether lumbosacral spinal cord epidural stimulation can promote functional standing in individuals with clinically motor and sensory complete (graded AIS A) classification. We had preliminary evidence from one AIS B participant that full weight-bearing standing with independence of leg extension could be achieved [20]. Therefore, we studied two individuals classified as AIS A and an additional AIS B to assess whether clinically detectable supraspinal sensory sparing was necessary to achieve standing without external assistance when epidural stimulation was provided. This study was also aimed to evaluate the role of weight-bearing related sensory information in modulating the EMG activity of the lower limbs’ muscles and identify the specific stimulation parameters needed to promote standing with the least amount of external assistance for hip and knee extension in the four research participants.

We hypothesized that: 1) clinically detectable supraspinal sensory sparing was not required to achieve full weight-bearing standing with epidural stimulation; 2) weight-bearing related sensory input projected to the spinal circuitry enabled the generation of EMG patterns sufficient for standing when epidural stimulation was provided; and 3) stimulation parameters similar to the ones that facilitated standing in the first AIS B participant could promote standing with the least amount of assistance also in the other three SCI individuals. The results of the present study have important implications with respect to: 1) how lumbosacral neural networks can be selectively modulated by varying the epidural stimulation parameters, and 2) identifying the most efficacious strategies for improved motor function for standing after sensory and motor complete paralysis.

## Materials and Methods

### Participants

Four individuals with chronic SCI, who met the following inclusion criteria, were recruited as participants of this study: (1) stable medical condition without cardiopulmonary disease or dysautonomia that would contraindicate standing or stepping with body weight support training; (2) no painful musculoskeletal dysfunction, unhealed fracture, contracture, pressure sore, or urinary tract infection that might interfere with stand or step training; (3) no clinically significant depression or ongoing drug abuse; (4) no current anti-spasticity medication regimen; (5) non-progressive spinal cord injury above T10; (6) AIS A or B; (7) no motor response present in leg muscles during trans-magnetic stimulation; (8) not present or bilateral delay of sensory evoked potentials; (9) no volitional control during voluntary movement attempts in leg muscles as measured by EMG activity; (10) segmental reflexes remain functional below the lesion; (11) brain influence on spinal reflexes is not observed as measured by EMG activity; (12) must not have received Botox injections in the previous 6 months; (13) be unable to stand or step independently; (14) at least 1-year post-injury; and (15) must be at least 18 years of age. The research participants signed an informed consent for electrode implantation, stimulation, and physiological monitoring studies approved by the University of Louisville and the University of California, Los Angeles Institutional Review Boards. The individuals in this manuscript have also given written informed consent (as outlined in PLOS consent form) to publish these case details. One of the four research participants, B07, was the subject of the previous case study [20] that investigated the effects of lumbosacral spinal cord epidural stimulation on the recovery of motor function.

This study is not a clinical trial as determined by the FDA and the funding sources including the NIH. This study is not consistent with the WHO definition in that the outcomes reported in this study are not health-related outcomes. They address basic research and studies of the role of sensory processing and spinal circuitry in standing. This study was not designed as an interventional treatment.

#### Clinical and neurophysiological evaluations

Clinical and neurophysiological evaluations were performed to assess motor and sensory status of the four research participants (Table 1). Prior to implantation, two clinicians independently performed a physical exam following the International Standards for Neurological Classification of Spinal Cord Injury [21,22] in order to classify the injury using the ASIA (American Spinal Injury Association) Impairment Scale (AIS). Individuals A45 and A53 had no sensory or motor function below the lesion including the sacral segments S4-S5, being classified as AIS A. Individuals B07 and B13 showed impaired sensory and no motor function below the neurological level of the lesion (AIS B).

**Table 1:** Clinical characteristics of individuals.

Participant	Age	Gender	Post injury	Neuro level	AIS grade	AIS score						Anal sensation
	yr		yr			Sensory (T10-S5, score out of 18)				Motor (lower extremity)		
						L LT	L PP	R LT	R PP	L	R	
B07	24	Male	3.4	T2	B	15	11	18	10	0	0	yes
A45	24	Male	2.2	T4	A	0	0	0	0	0	0	no
B13	33	Male	4.2	C7	B	10	10	10	8	0	0	yes
A53	27	Male	2.3	T4	A	0	0	0	0	0	0	no

Neuro level: neurological level of the lesion; AIS: American Spinal Injury Association (ASIA) Impairment Scale. Sensory score was designated by light-touch (LT) and pinprick (PP) of the left (L) and right (R) lower limb, below the lesion.

Upper and lower extremity somatosensory evoked potentials, transcranial magnetic stimulation (TMS) and residual motor output were also assessed during various maneuvers [23–28] before and after implantation, without epidural stimulation, as reported in details by Angeli et al. [29]. In summary, when the median nerve was stimulated at the wrist, all participants showed normal somatosensory evoked potentials. When lower extremity was stimulated at the posterior tibial nerve and ankle, both AIS-A individuals showed no response, whereas both AIS-B individuals showed bilateral cortical delays as previously reported. TMS was used to assess the functional integrity of the cortico-spinal tracts [30,31] in three out of the four individuals (B07, A45 and A53). No motor evoked potentials from left and right soleus and tibialis anterior muscles were detected during TMS when participants were asked both to relax and to attempt a sustained dorsiflexion. In all individuals, EMG activity during attempts to move the lower limbs and during reinforcement maneuvers was similar to the EMG recorded during relaxation. Also, prior to implantation, after 80 sessions of Locomotor Training (combined stand and step training, with stepping comprising the majority of minutes [32]) there were no significant changes in the EMG activity during assisted stepping in any of the four research participants. In summary, without epidural stimulation, no functional motor connectivity between the supraspinal and spinal centers below the level of injury was detected in any of the four research participants.

#### Surgical implantation of electrode array and stimulator

The epidural spinal cord stimulation unit (Restore ADVANCED, Medtronics) was used to electrically stimulate the lumbosacral enlargement. A 16-electrode array (5-6-5 Specify, Medtronics) was implanted at vertebral level T11-L1 over the spinal cord segments L1-S1 in all individuals. The electrode lead was tunneled to a subcutaneous abdominal pouch where the pulse generator was implanted.

#### Experimental Procedures

Research participants performed experimental and training sessions for standing using a custom designed standing frame comprised of horizontal bars anterior and lateral to the individual. These bars were used for upper extremity support and balance assistance as needed. If the knees or hips flexed beyond the normal standing posture, external assistance was provided at the knees distal to the patella to promote extension, and at the hips below the iliac crest to promote hip extension and anterior tilt. Facilitation was provided either manually by a trainer or by elastic cords, which were attached between the two vertical bars of the standing apparatus. Mirrors were placed in front of the participant and laterally to him, in order to allow a better perception of the body position via visual feedback.

Stimulation began while the participant was seated. Then the participant initiated the sit to stand transition by positioning his feet shoulder width apart and shifting his weight forward to begin loading the legs. The participant used the horizontal bars of the standing apparatus during the transition phase to balance and to partially pull himself into a standing position. Trainers positioned at the pelvis and knees manually assisted as needed during the sit to stand transition.

#### Stand training

After the stimulator implantation, research participants underwent 80 sessions of stand training (1 hour, 5 sessions per week). Stand training was always performed with lumbosacral spinal cord epidural stimulation. Participants were encouraged to stand for as long as possible throughout the training session, with the goal to stand for 60 minutes with the least amount of assistance. Seated resting periods occurred when requested by the individuals. Rest needed varied across participants. The data reported in this study were recorded after all sessions of stand training were concluded.

#### Stimulation parameters

Stimulation parameters were initially selected during sitting with the intent to promote an enabling-stimulation scenario rather than imposing a motor output by stimulation alone (without weight-bearing related sensory information). If standing without external assistance for hip and knee extension was not achieved with the initial setting, a protocol to modify stimulation parameters during upright posture was followed in order to seek improvements of motor function for standing.

### a) Initial stimulation parameters selected during sitting.

- a near-motor threshold stimulation amplitude that did not elicit directly lower limb movements;
- a stimulation frequency of 25 Hz, because it was found as effective for standing as 15 Hz in the first subject (B07) of the previous case study [20], and because relatively higher frequencies were reported to promote greater activation of interneurons [19,33];
- a wide-field electrode configuration with cathodes positioned caudally, because it evoked nonlocation-specific responses in both proximal and distal muscles [34], and because cathodes positioned caudally were shown to possibly promote motor patterns characteristic of standing behaviour [17] in clinically motor complete SCI while lying supine.

### b) Improvement of stimulation parameters.

If the initial stimulation parameters did not promote EMG patterns sufficient to full body weight-bearing standing without external assistance for hip and knee extension, the following guidelines were adopted to modify stimulation parameters in order to improve motor function.

- Stimulation frequency and amplitude were modulated synergistically in order to find the higher stimulation frequency that elicited an overall continuous (non-rhythmic) EMG pattern effective to bare body weight.
- Specific electrode configuration adjustments were defined to seek improvements of different aspects of motor output (Table 2). The rationale for these changes is related to both previous findings reported in the literature and results of previous experiments performed on the same research participants in supine position with different bipolar and wide field electrode configurations (as partially reported by Sayenko and colleagues [34]), which provided individualized maps of motor pools activation. These guidelines were used to determine which electrode configurations, out of those potentially available (~4.3*10^7 combinations of electrodes), were to be examined in order to seek improvements of motor function for standing. Weekly experimental sessions were performed to monitor standing behaviour and EMG from lower limb muscles with adjusted stimulation parameters to contribute to the selection of such parameters following the same protocol.

**Table 2:** Guidelines adopted to define which electrode configuration adjustments were to be tested in order to improve different aspects of motor output during standing.

Motor output improvement	Electrode configuration adjustment
To compensate activation differences between left and right lower limb.	To unbalance anodes and cathodes between the lateral columns of the electrode array. [10]
To focus the stimulation on predominant extensor activation.	To adjust cathodes position in order to target primarily extensors muscle groups, according to the individualized map of motor pools activation.
To find common stimulation amplitude that activates distal and proximal muscle groups simultaneously.	To narrow the electrode field to the caudal portion of the electrode array in order to increase the excitability of distal muscles’ motoneuron pools, or to extend the electrode field toward the rostral portion of the array in order to increase the excitability of proximal muscles’ motoneuron pools. [34]

#### Data Acquisition and analysis

EMG and ground reaction forces data were recorded at 2000 Hz using a custom-written acquisition software (National Instruments, Austin, TX). EMG activity of right (R) and left (L) gluteus maximus (GL), medial hamstring (MH), rectus femoris (RF), vastus lateralis (VL), tibialis anterior (TA), medial gastrocnemius (MG) and soleus (SOL) was recorded by means of bipolar surface electrodes with fixed inter-electrode distance [20]. Bilateral EMG from the iliopsoas (IL) was recorded with fine-wire electrodes. Two surface electrodes were placed symmetrically lateral to the electrode array incision site over the paraspinal muscles in order to record the stimulation artefacts, which were used as indicators of the stimulation onset (time points when the stimulus pulses were applied). The time between stimulation onset and the EMG response onset was defined as the latency time of the evoked response. The amplitude of spinal cord evoked responses was quantified by peak to peak amplitude. The differences in amplitude were statistically evaluated by Student’s paired t test. To investigate the variability of the spinal cord evoked responses generated at different stimulation frequencies, the coefficient of variation (standard deviation / mean) was calculated over 20 ms after the onset of the spinal cord evoked responses (N = 20), which were selected within a representative portion of continuous (not rhythmic) EMG recording. Ground reaction forces were collected using a high-resolution pressure sensing mat (HR mat system, TEKSCAN, Boston, MA).

## Results

### Individual-specific stimulation parameters promoted full body weight-bearing standing in clinically complete SCI participants

**Fig 1:**
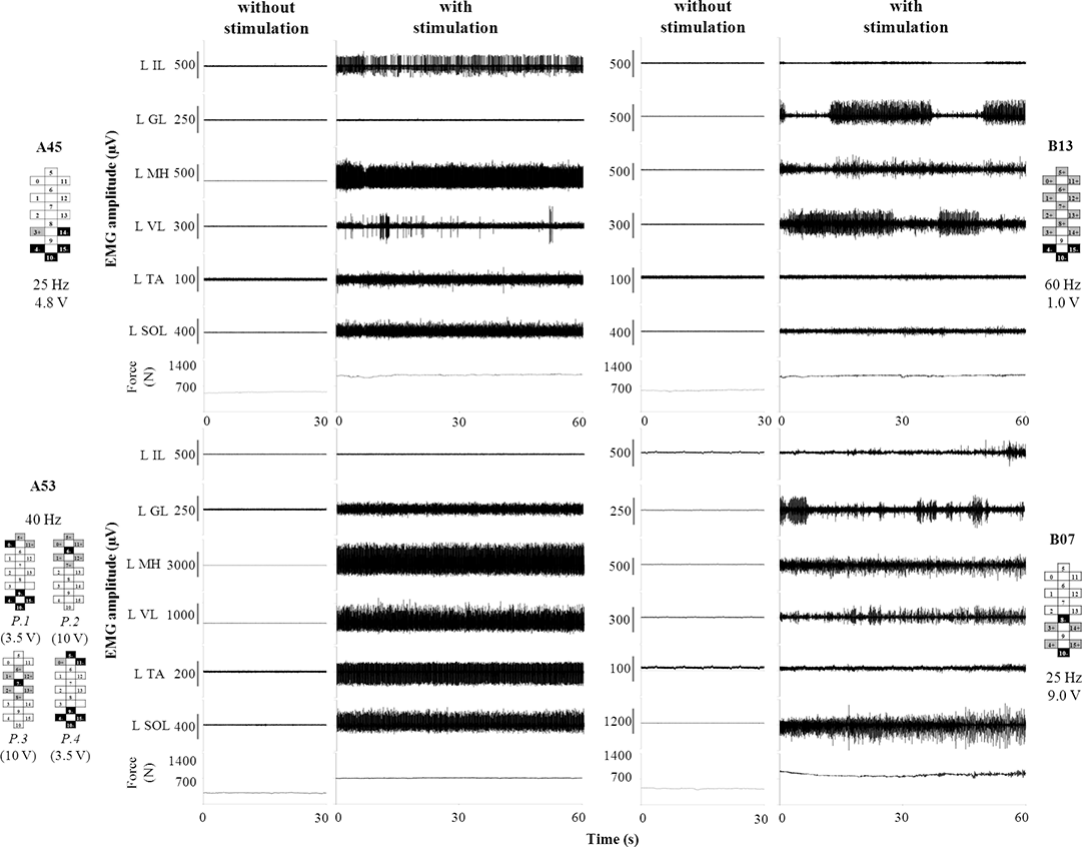
EMG and ground reaction forces recorded during full weight-bearing standing. Time course of EMG and ground reaction force recorded during representative standing without stimulation and with stimulation parameters that promoted standing with the least amount of assistance. Participants A45 and A53 were able to stand placing their hands on the horizontal bars of the standing apparatus to assist balance. Participants B07 and B13 also used elastic cords fixed to the apparatus to assist with hip extension (S1 Video). Stimulation frequency, amplitude and electrode configuration (cathodes in black, anodes in grey, and non-active in white) are reported for each participant. Participant A53 was stimulated with four programs (P.1 to P.4) delivered sequentially at 10 Hz, resulting in an ongoing 40 Hz stimulation frequency. IL: iliopsoas; GL: gluteus maximus; MH: medial hamstring; VL: vastus lateralis; TA: tibialis anterior; MG: medial gastrocnemius; SOL: soleus.

Four out of four research participants achieved full weight-bearing standing with minimal self-balance assistance when their specific stimulation parameters were used to stimulate the lumbosacral spinal cord (S1 Video, Fig 1). The two clinically sensory and motor complete participants (A45 and A53) were able to stand without any external assistance, placing their hands on the horizontal bars of the apparatus to assist balance. The other two participants (B07 and B13) also used elastic cords fixed to the standing frame to assist with hip extension. Representative EMG patterns recorded from the four participants during stable standing are shown in Fig 1. EMG activity was overall continuous (not rhythmic) and modulated over time in some muscles (i.e. VL in participant A45; GL and VL in participant B13; GL in participant B07). Standing could be achieved with a variety of EMG patterns. For example, MH and SOL were most consistently active in all participants, while the EMG activity of TA was little or negligible in B13 and B07. Also, IL was consistently active in A45 but not active in A53. Conversely, without stimulation, little or no EMG activity was recorded from the analysed muscles of all research participants. In this case, research participants achieved and maintained upright posture because of the trainers’ assistance at the knees and hips, and because of the weight-bearing action performed by the participants’ upper limbs. The greater amount of assistance during standing without stimulation resulted in the lower ground reaction forces (-46.9 ± 6.7%) as compared to standing with stimulation.

**Fig 2:**
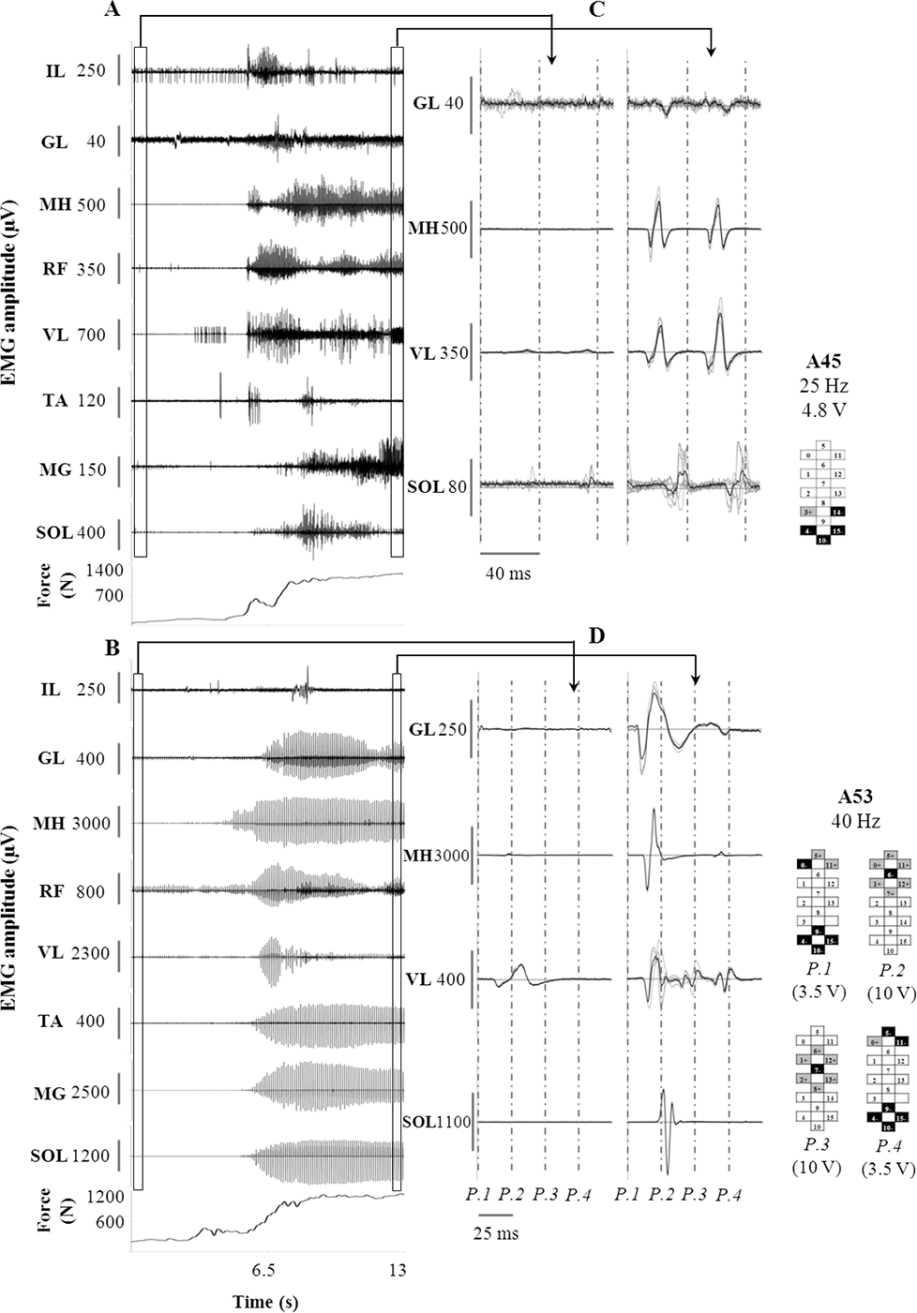
EMG and ground reaction forces during sitting to standing transition. Time course of EMG and ground reaction force recorded during sitting to standing transition from participants A45 (Panel A) and A53 (Panel B). Panels C and D: Spinal cord evoked responses taken from the windows entered in A and B, respectively (left window: sitting; right window: standing). The black trace is the average of 15 spinal cord evoked potentials represented in grey. Vertical grey dotted line: stimulation onset. Stimulation frequency, amplitude and electrode configuration (cathodes in black, anodes in grey, and non-active in white) are reported. Participant A53 was stimulated with four programs (P.1 to P.4) delivered sequentially at 10 Hz, resulting in an ongoing 40 Hz stimulation frequency. IL: iliopsoas; GL: gluteus maximus; MH: medial hamstring; RF: rectus femoris; VL: vastus lateralis; TA: tibialis anterior; MG: medial gastrocnemius; SOL: soleus.

The sensory information related to the transition from sitting to standing (S2 Video) remarkably modulated the EMG activity of lower limb muscles. Representative examples of EMG and force data recorded from A45 and A53 showed little or no EMG during sitting (Fig 2A and 2B, respectively); the transition of weight onto the lower limbs promoted a significant increase in the level of EMG activity. Spinal cord evoked responses were not detected in some muscle during sitting (see MH in Fig 2C and SOL in Fig 2D as examples), while they were recorded in all muscles during standing. The amplitude of spinal cord evoked responses was greater in standing than in sitting for all participants and investigated muscles, with some exception showed by TA and IL (S1 Fig). Such EMG modulation occurred during the sitting to standing transition without any change in the stimulation parameters, which were the same delivered to the research participants’ spinal cord in order to achieve full weight-bearing standing with minimal assistance (Fig 1). These stimulation parameters were substantially different among individuals: frequency and amplitude ranged from 25 to 60 Hz and from 1.0 to 9.0 V, respectively. Electrode configurations generally included the placement of cathodes in the caudal portion of the array and an individual-specific assignment of other electrodes (both anodes and cathodes), resulting in configurations dissimilar among research participants.

**Fig 3:**
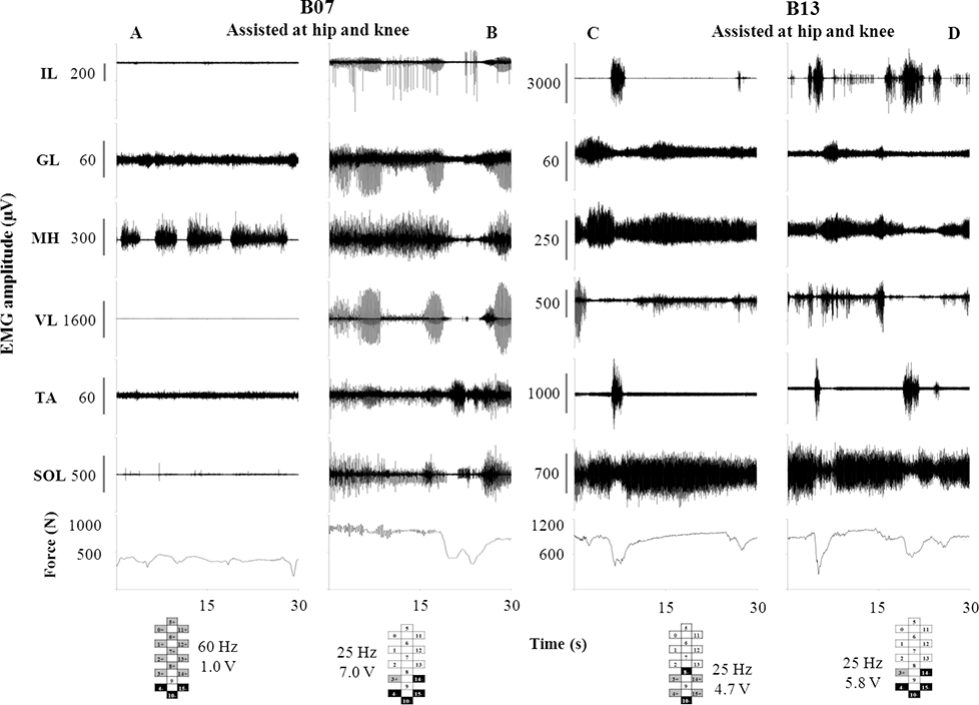
EMG and ground reaction forces recorded during standing with stimulation parameters optimal for other individuals. Time course of EMG and ground reaction force recorded from participants B07 (Panels A and B) and B13 (Panels C and D) during standing with stimulation frequency and electrode configuration optimal for other participants: A, specific for B13; B and D: specific for A45; C: specific for B07. Stimulation amplitude was adjusted to optimize standing. External assistance to maintain hip and knee extension was needed to stand in all four conditions. IL: iliopsoas; GL: gluteus maximus; MH: medial hamstring; VL: vastus lateralis; TA: tibialis anterior; SOL: soleus.

Stimulation configurations optimized for one individual did not generate sufficient EMG patterns to support full weight-bearing standing in the other research participants. For example, when participant B07 was stimulated with parameters optimized specifically for B13, strong rhythmic EMG was observed in the MH, with very low levels of continuous activity in all other muscles; this motor pattern resulted in the need of external assistance to maintain hip and knee extension (Fig 3A). The average ground reaction force (395 ± 40 N) showed that at least 50% of his body weight was supported by external assistance. Stimulation parameters specific for A45 induced in all muscles greater EMG activity and higher ground reaction forces (696 ± 141 N) than the parameters specific for B13 (Fig 3B). However, the EMG pattern was predominantly rhythmical in IL, GL and VL, leading to instability and need of external assistance for standing. Stimulation parameters optimized specifically for B07 and A45 also induced rhythmic EMG patterns in participant B13 (Fig 3C and 3D, respectively). High amplitude EMG bursts of IL and TA coincided with a steep decrease in ground reaction force due to the loss of knee extension, resulting in a greater level of external assistance needed to maintain hip and knee extension. Similarly, stimulation parameters optimized specifically for B07, B13 and A45 promoted EMG patterns that were not sufficient to support standing without external assistance at hips and knees in participant A53 (S2 Fig).

### Effects of electrode configurations on EMG characteristics

**Fig 4:**
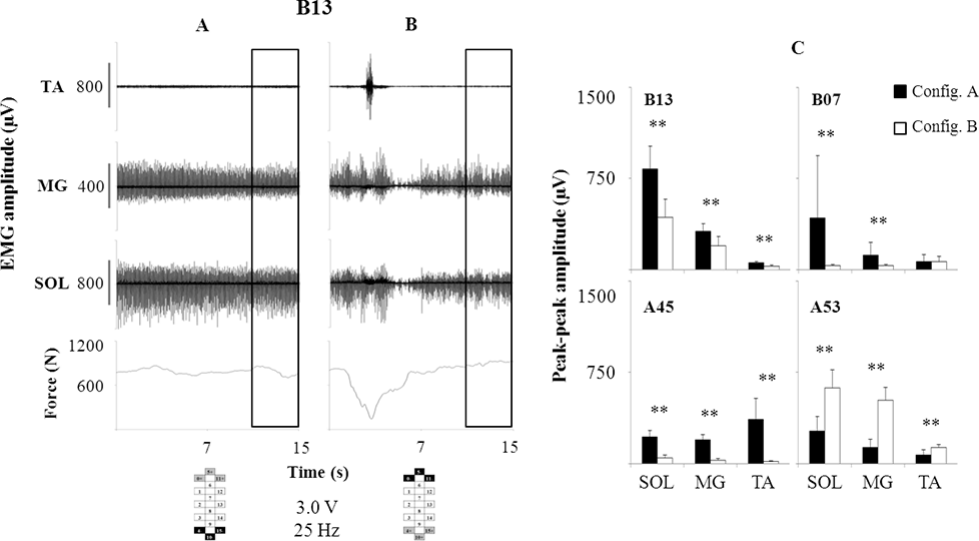
EMG and ground reaction forces recorded during standing with different electrode configurations. EMG and ground reaction force were recorded from all participants during standing. Stimulation amplitude and frequency (3.0 V and 25 Hz, respectively) were delivered with a wide-field electrode configuration. Cathodes were placed either in the caudal (Panel A) or rostral (Panel B) portion of the array; anodes were placed specular to cathodes, as shown in the bottom left of the figure.Panel C: Average (N = 100) peak to peak spinal cord evoked potentials amplitude recorded from the four participants during standing with cathodes placed caudally (black bars, Configuration A) or rostrally (white bars, Configuration B). As for participant B13, spinal cord evoked potentials were taken from the windows entered in A and B. TA: tibialis anterior; MG: medial gastrocnemius; SOL: soleus.

The cathodes (active electrodes) placed in the caudal portion of the electrode array and more caudally than the anodes (reference electrodes) generally promoted a more effective EMG pattern for standing. When the relative position of cathodes and anodes was studied within a wide field multipolar electrode configuration, external assistance to facilitate hip and knee extension was needed to stand in all participants with both configurations (Fig 4). However, a more continuous EMG pattern was generally recorded from the leg muscles with cathodes positioned in the caudal portion of the array; this was accompanied by higher EMG amplitude in three out of four research participants. The effectiveness of the cathodes placed more caudally than the anodes is even more pronounced in the case of participant A45 with the stimulation focused on the caudal portion of the lumbosacral spinal cord (Panel A in S3 Fig), promoting continuous EMG activity and standing without external assistance. The inversion of cathodes and anodes resulted in rhythmic EMG activity and modulation of EMG amplitude (i.e. greater for VL; lower for MH and SOL; Panel B in S3 Fig). In this case, external assistance at hips and knees was needed to stand, and the attempt was interrupted because of the discomfort caused by the stimulation (abdominal contractions). The distribution of anodes and cathodes along the lateral columns of the electrode array could also substantially impact EMG pattern and standing behaviour (Panels C and D in S3 Fig; S3 Video). For example, when the same caudal portion of the lumbosacral spinal cord was stimulated with anodes and cathodes unbalanced in the eighth row (from the top) of the electrode array, EMG pattern was still overall continuous and standing was achieved without external assistance. However, the lateral anode-cathode unbalance of both eighth and tenth rows of the electrode array resulted in additional assistance needed to stand (at knees and hips) and discomfort (abdominal contractions). EMG bursts also occurred in MH and VL, as well as a reduction of SOL EMG amplitude.

### Interplay between stimulation amplitude and frequency

**Fig 5:**
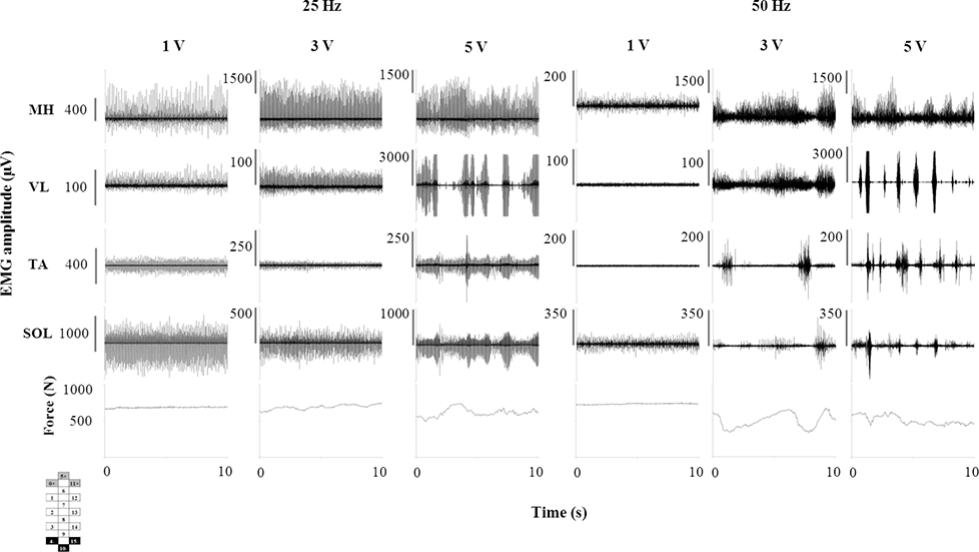
EMG and ground reaction force recorded during standing with different stimulation amplitudes. EMG and ground reaction force recorded from participant A53 during standing with three different stimulation amplitudes (1.0, 3.0 and 5.0 V) delivered at either 25 Hz or 50 Hz. Electrode configuration (cathodes in black, anodes in grey, and non-active in white) is reported. MH: medial hamstring; VL: vastus lateralis; TA: tibialis anterior; SOL: soleus.

**Fig 6:**
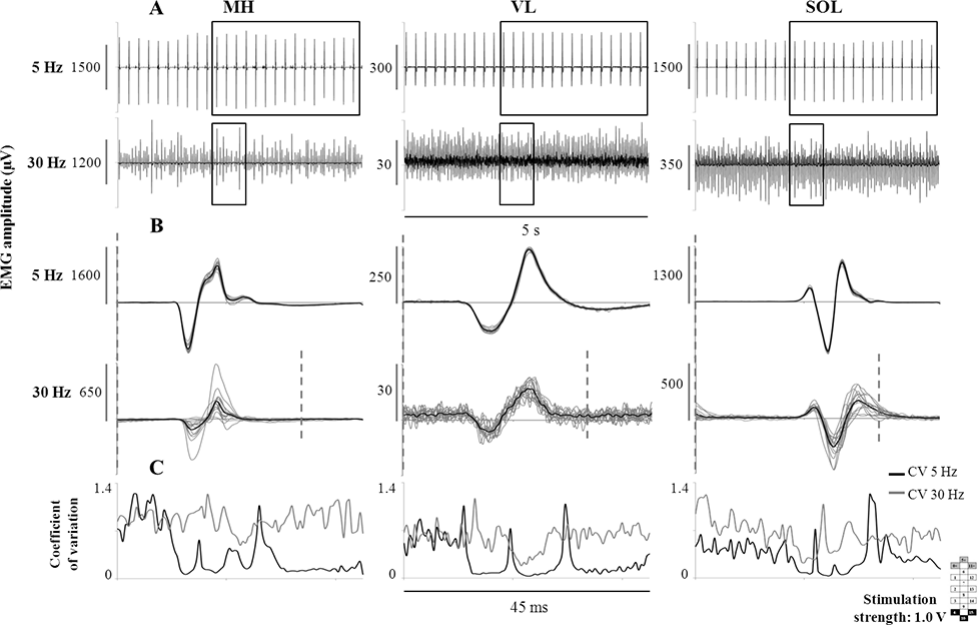
EMG recorded during standing at lower and higher stimulation frequency. Panel A: Continuous EMG pattern recorded from participant A53 during standing at lower (5 Hz) and higher (30 Hz) stimulation frequency. Panel B: Spinal cord evoked responses taken from the windows entered in A. The black trace is the average of 20 responses represented in grey. Vertical grey dotted line: stimulation onset. Panel C: Coefficient of variation (CV) calculated from the spinal cord evoked responses reported in B (black line: 5 Hz; grey line: 30 Hz). Stimulation amplitude and electrode configuration (cathodes in black, anodes in grey, and non-active in white) are reported. MH: medial hamstring; VL: vastus lateralis; SOL: soleus.

EMG activity and standing behaviour were highly responsive to the interplay between stimulation amplitude and frequency. When the spinal cord was stimulated with higher frequencies (i.e. 25 Hz and 50 Hz), the EMG pattern generated depended on the stimulation amplitude. Lower level of amplitude (1.0 V) promoted continuous EMG activity in all muscles, a constant level of ground reaction forces and the independent extension of the left knee (at 25 Hz only) (Fig 5). At 25 Hz, the EMG pattern remained continuous when the amplitude increased to 3.0 V; conversely, EMG bursts and unstable standing behaviour were shown at 50 Hz. Higher level of amplitude (5.0 V) induced rhythmic EMG activity in several muscles at 25 Hz and 50 Hz, as well as unstable standing behaviour. Also, when overall continuous EMG patterns were promoted during standing (Fig 6A), lower stimulation frequency induced spinal cord evoked responses very consistent in shape and amplitude (Fig 6B). At higher frequency, amplitude and shape of the spinal cord evoked responses varied substantially over time. The greater variability induced by higher frequency was quantified by the greater coefficient of variation (Fig 6C), which tended to increase as the stimulation frequency increased from 2 to 30 Hz, in all participants and investigated muscles (S1 Table).

## Discussion

We showed that sensory and motor complete SCI individuals were able to achieve full weight-bearing standing without any external assistance and with only minimal self-balance assistance provided by their hands when the lumbosacral spinal cord was epidurally stimulated. Also, we have highlighted the importance to select individual-specific parameters in order to achieve standing with the least amount of assistance: stimulation parameters optimized for one individual resulted in poor standing and additional need of external assistance for hip and knee extension in the other participants. Stimulation parameters were crucial determinants of the motor patterns generated during standing by the human spinal cord. Electrode configurations with cathodes placed in the caudal portion of the array, and more caudally than the anodes, generally induced continuous EMG activity, higher level of activation of leg muscles and better standing behaviour. Also, at higher stimulation frequencies, the EMG pattern of several muscles changed from continuous to rhythmic as the stimulation amplitude increased. Furthermore, as previously reported in one individual [20], we have confirmed that weight-bearing related sensory information projected to the spinal cord was essential to generate sufficient EMG patterns to effectively support full weight-bearing standing when epidural stimulation was provided.

### Lumbosacral spinal cord epidural stimulation and sensory information enabled the participants to regain full body weight-bearing standing

Our results suggest that full body weight-bearing standing was enabled by epidural stimulation, which modulated the lumbosacral neural networks to a functional state that optimized the integration of task-specific afferent input to result in the generation of effective motor patterns. All four participants with motor complete paralysis achieved full weight-bearing standing with minimal self-balance assistance and without any external assistance to facilitate knee extension, showing an overall continuous EMG pattern of the lower limb muscles and constant level of ground reaction forces only when epidural stimulation was provided (Fig 1). In particular, the two clinically sensory and motor complete individuals (A45 and A53) showed an overall improved standing ability as compared to the first participant that was previously studied (B07, [20]), because they were able to stand without any external facilitation to promote hip extension. This ability was not related to a common activation pattern of the main hip flexor and extensor analysed (IL and GL, respectively), which was different between these two participants (Fig 1). Further studies are needed to investigate which other lower limb and/or trunk muscles, if any, played a role in achieving standing without hip assistance. However, this finding supports the hypothesis that clinically detectable sensory input from the lower limbs to the supraspinal structures was not required to achieve full weight-bearing standing without external assistance when lumbosacral epidural stimulation was provided, thus supporting a primary role of sensory information projected to the human spinal circuitry for eliciting motor patterns sufficient for standing.

In all four participants, the EMG activity was significantly modulated when the sitting to standing transition occurred and the loading of the legs was initiated, without any change in the stimulation parameters (Fig 2, S1 Fig). Motor patterns sufficient to elicit standing behaviour were generated when the lower limbs were loaded while transitioning from flexion (during sitting) to extension (during standing), indicating that the related sensory information was integrated by the spinal circuitry to result in the complex motor pool activation showed during standing. This finding supports our hypothesis, in that epidural stimulation itself was not directly inducing motor pool activations appropriate for standing. The lumbosacral circuitry of the research participants was considered functionally isolated from supraspinal influence since they were classified as motor complete (AIS A and B classifications). In addition, all other neurophysiological assessments performed without lumbosacral epidural stimulation did not indicate functional connectivity across the injury level [29] suggesting that any of the above mentioned EMG modulation would be predominantly generated at the level of the human spinal cord. However, we cannot exclude that supraspinal influences contributed to achieve standing, since these four research participants were able to voluntarily move their legs when the lumbosacral circuitry was stimulated with specific stimulation parameters [29]. On the other hand, they were not able to voluntarily move their legs when the stimulation parameters that promoted standing with the least amount of assistance were applied.

The comparison of EMG amplitude recorded from sitting and standing might be affected by possible variability due to the relationship of the epidural electrode array with the spinal cord. In particular, the cord may slide inside the dura, and hence the relative position of the cord with respect to the electrode array may vary if the anatomical position of the spine changes. In order to minimize the possible impact of this issue, participants were asked to sit and to stand with the trunk in a straight standardized position. However, functional load through weight-bearing has been widely shown to play a significant role in modulating the motor pattern generated during stepping [11,35–39] and standing with and without epidural stimulation in mammals. Decerebrated and spinalized cats showed the ability to exert efficient postural control during standing and stepping in the presence of epidural stimulation [40,41]. In these studies, the suppression of vestibular, visual, and head-neck-trunk sensory input implies that the motor responses were driven by somatosensory information from the lower limbs, highlighting the importance of afferent information in modulating motor output. Spinal rats also recovered standing when appropriate epidural stimulation sites and frequencies were selected, and a modest level of stimulation amplitude (primarily sub-motor threshold) was delivered to the spinal cord [8]. Thus, motor responses in standing were not directly imposed by stimulating at high intensities; rather they were facilitated and modulated by the ensemble of sensory information being projected to the spinal circuitry, the physiological state of which was properly modulated by the stimulation.

### Mechanisms of lumbosacral spinal cord epidural stimulation

The mechanisms underlying the enhancement of the spinal circuitries by epidural stimulation are not yet completely understood [6]. Nevertheless, neurophysiological recordings suggest that spinal cord epidural stimulation engages spinal circuits mainly by recruiting dorsal root fibres carrying somatosensory signals from the limbs at their entry into the spinal cord as well as along the longitudinal portions of the fiber trajectories [42–44]. Regarding the pathways that can be modulated by epidural stimulation, it is important to recognize the role of both complex spinal neural connections and stimulation parameters. Previous studies reported that most muscles are innervated by several spinal segments, and that network of motoneurons appears to be broadly spaced over wide regions of the spinal cord [45,46]. In addition, extensive divergence of a single Ia fiber from each muscle spindle showed extensive synaptic connectivity to the homonymous motor pools as well as to synergists and, indirectly, to antagonistic motor pools through Ia inhibitory interneurons [47]. Also, intersegmental connectivity among the lumbar segments via ascending projections from the sacral segments has been reported [48]. Combined, these observations are consistent with the interpretation that epidural stimulation impacts many different sensory-motor pathways simultaneously, even if a relatively localized stimulation is used [34]; however, the stimulation parameters (i.e. frequency, amplitude, site) are crucial determinants of the extent and proportion of the modulation of these pathways [8].

#### Stimulation frequency and amplitude

Dimitrijevic and colleagues elicited lower limb extension in the supine position using epidural stimulation of the lumbosacral spinal cord with two active electrodes of a quadripolar array in individuals with motor complete spinal cord injury who were implanted with the intent to reduce spasticity [16–19]. In particular, they reported that frequencies between 5 and 15 Hz were optimal to initiate and retain tonic activation of leg muscles resulting in extension behaviour while lying supine [17]. The related short latency EMG evoked potentials were timed to the stimulation frequency and the amplitudes of the hamstring and triceps surae were significantly higher than the quadriceps and tibialis anterior, respectively, without any external loading through the sole of the feet. They also reported that at higher frequencies (21–31 Hz), without any adjustment in stimulation site and intensity, the EMG pattern became rhythmic and the relative amplitude of the flexors and extensors were then reversed.

They interpreted these results as the different stimulation frequencies at the same site of stimulation would access different inhibitory and/or excitatory pathways within spinal cord networks to elicit different EMG patterns (i.e. rhythmic vs tonic). Thus, at frequencies between 5–15 Hz the interneuronal network was conceivably configured via presynaptic and synaptic mechanisms to favour the EMG “extensor pattern”, and at higher frequencies to favour a rhythmic locomotor-like pattern. We were also able to generate motor responses similar to those reported by Dimitrijevic and colleagues at lower stimulation frequencies (i.e. 5 Hz), with spinal cord evoked responses consistent in shape and amplitude (Fig 6B) during weight-bearing standing with external assistance. However, the motor behaviour at lower frequencies was ineffective for standing because of the pulsatile muscle contractions elicited, linked to the stimulation frequency. Further, independence from external assistance was predominantly observed at higher frequencies, in fact, similar to those frequencies in their studies that resulted in rhythmic patterns in the supine position without leg loading [6,16,18,19]. In contrast, we selected below or near-motor threshold amplitudes (Fig 2) whereas in the above studies the amplitude was set well above motor threshold. When we increased the stimulation amplitude we also observed rhythmic EMG activity (Fig 5) with consequent impairment of standing behaviour. These differences in stimulation methodology and the greater flexibility allowed by our longer and more complex array most likely contributed to the differences between the studies.

The role of stimulation amplitude on the modulation of sensory-motor pathways has been also investigated in three of the four research participants of this study during experiments in supine position [34]. The outcomes suggested that lower stimulation amplitudes resulted in initial recruitment of the lower threshold afferent structures, while with higher amplitudes more efferent volleys are involved, precluding the response that may have been driven by the afferent pathways and leading to the activation of motoneurons and/or anterior roots.

In view of the previous findings reported in the literature, it can be hypothesized that the relatively lower stimulation amplitudes and higher frequencies that promoted standing in the present study, rather than driving directly extensor patterns, altered the excitability of the spinal circuitry allowing the load bearing related sensory information to drive the circuitry to generate extensor motor patterns that supported standing without external assistance (Fig 2). Conceivably, additional afferents (i.e. Ib afferents from Golgi tendon organs, group II secondary muscle afferents and cutaneous afferents) progressively contributed to shape the spinal cord evoked responses through the activation of interneurons when relatively higher stimulation frequencies were used [19,33]. This would contribute to explain the greater variability of the spinal cord evoked responses observed at higher as compared to lower frequencies (Fig 6; S1 Table) and, possibly, the achievement of standing with the least amount of assistance at relatively higher frequencies (Fig 1).

#### Electrode configuration

Stimulation site was shown to be crucial for promoting different motor functions after complete SCI. Some evidence suggested that L2 spinal level is the most responsive to epidural stimulation for inducing locomotor-like activity in rats and humans [6]. In rats, one study showed that rostral stimulation (cathode and anode at L2 and L5 spinal level, respectively) promoted body weight-bearing standing, whereas more caudal stimulation failed to facilitate standing [8]. However, another study indicated facilitation of motor function for standing when the stimulating electrode was placed caudally (spinal segment S1), whereas a more rostral stimulation site (spinal segment L2) induced a rapid and prolonged flexion of the hindlimb [10]. Previous spinal cord epidural stimulation studies in humans [16–19,49,50] provided some evidence that cathodes positioned caudally could promote motor patterns characteristic of standing behaviour. In particular, lower limb extension patterns were induced in individuals with SCI and similar neurophysiological characteristics as in the present study while lying supine, when the stimulation site encompassed from the levels of the spinal cord L2/3 to L5, and in one case as low as S1/2 [17]. Electrode configurations with the cathode (active electrode) more caudal than the anode always resulted in lower thresholds for activation, leading the authors to surmise that potentially the lower rather than the upper lumbosacral spinal cord would be optimal to generate extension pattern.

Our results also support the view that the activation of lower portion of the lumbosacral spinal cord (L4-S1) is more effective in facilitating a lower limb extension pattern than the upper lumbar cord. We observed that in essentially all comparisons more continuous EMG and higher amplitudes of plantar flexors with an overall more stable weight-bearing standing behaviour was induced when the electrodes assigned as a cathode were more caudal than those assigned as an anode (Fig 4; Panels A and B in S3 Fig). However, the longer electrode field (46.5 mm) over the spinal-cord segments L1–S2 did not promote standing without external assistance (Fig 4). Conversely, standing with the least amount of assistance was achieved with different electrode configurations across individuals (Fig 1), although active electrodes (cathodes) were consistently placed in the caudal portion of the array. These individual-specific electrode configurations resulted from different adjustments tested during standing, because the sensory-motor pathways targeted by stimulation are reconfigured by the execution of a specific motor task [10]. These adjustments were performed taking into consideration the individualized map of motor pools activation, in order to target primarily extensors muscle groups, and previous findings reported in the literature that showed the effects of stimulation site on topographical motor pools recruitment in spinal mammals. For example, in case of activation differences between proximal and distal muscles that were potentially responsible for impaired standing, we attempted either to narrow the electrode field to the caudal portion of the electrode array or to extend the electrode field toward the rostral portion of the array in order to increase the excitability of distal or proximal muscles’ motoneuron pools, respectively [34]. However, these changes influenced the length of the electrode field, thus having implications also for the extent and proportion of the modulation of intersegmental efferent structures.

Similarly, if activation differences between the left and right lower limb were present, we tested the effects of unbalanced anodes and cathodes between the lateral columns of the electrode array with the intent to alter the side-specific limb muscles recruitment, seen as the medio-lateral positioning of the electrode influenced selectively the recruitment of side-specific limb muscles in complete rats [10]. However, the effects of even minimal electrode configuration modification were difficult to predict accurately. Indeed, the change in state of a single electrode of the lateral columns of the array could either maintain the same level of external assistance and a similar motor pattern (Panel A vs C in S3 Fig) or substantially influence standing behaviour and motor output (Panel C vs D in S3 Fig; S3 Video).

Research participant A53 achieved his best standing when four stimulation programs (each one with different electrode configuration and amplitude) were delivered sequentially at 10Hz to effectively result in an ongoing 40Hz stimulation. The uniqueness of the four interleaving program configuration overcame some of the challenges associated with activation differences between left and right side and among crucial muscles groups when stimulating at the same amplitude. Program one and program four (to a lesser extent) provided the activation of both proximal and distal muscles; however there was a lack of bilateral symmetry and hip extension if only one program was used. Programs two and three were designed to selectively target specific muscle groups (GL and knee extensors, respectively) that were otherwise weakly or not active.

The observation that stimulation parameters optimized for one individual did not promote sufficient EMG patterns to support full weight-bearing standing in the other participants (Fig 3; S2 Fig) was an unexpected challenge. Spinal cord anatomy and the position of the electrode array with respect to the spinal cord may have played a role in such individual-specific motor responses. Motor neuron density might exhibit inter-individual variability [45,46]; moreover, several muscles are innervated by and project sensory information to excitatory and inhibitory neurons associated with multiple motor pools, likely increasing such variability. Also, the loss of motor input following SCI leads to a reorganization of the underlying spinal circuitry, including interneuronal function [51–54]. It is likely that individual characteristics of the lesion and following plasticity of the spinal neural circuitry, among many, are factors that may have contributed to the different motor responses across research participants induced by stimulation parameters optimized for one individual. Further investigations on this topic are important for the development of epidural stimulation as a tool to improve the recovery of motor function after SCI.

Recently, improvement in stimulation technology resulted in a high-density electrode array that allowed different bipolar electrode configurations to be studied in spinal rats [8]. The related results underlined the importance of electrode location and anode–cathode orientation to generate EMG patterns effective for weight-bearing standing and stepping. Similar experimental models and also computational models [10] that investigate the effects of multi-electrode configurations as well as multiple-interleaving stimulation programs similar to those that promoted standing with the least amount of assistance in the present study would be valuable to take further advantage of such technology.

## Summary and clinical considerations

In conclusion, two clinically sensory and motor complete individuals, as well as two clinically motor complete and sensory incomplete individuals, achieved full weight-bearing standing with independent knee extension using minimal self-balance assistance when individual-specific stimulation parameters were delivered to the lumbosacral spinal cord via epidural stimulation. Weight-bearing related sensory information was essential to generate sufficient EMG patterns to support full weight-bearing standing in research participants with and without clinically detectable supraspinal sensory sparing. These findings highlight the potential of the human spinal circuitry and its capability to generate motor patterns effective for standing in the absence of functional supraspinal connections when epidural stimulation is provided. However, the intrinsic complexity of the spinal circuitry reorganized after a clinically complete spinal cord injury requires individualized stimulation parameters in order to finely modulate the sensory-motor pathways impacted by epidural stimulation and generate motor output effective for standing. Further efforts should be addressed to the study of mechanisms underlying the generation of these motor patterns, as well as the effects of training and more sophisticated and dedicated stimulation technology, in order to optimize the use of intrinsic lumbosacral circuits in recovering of motor functions for standing after SCI.

From a clinical perspective, it seems more feasible to modulate the physiological state of the lumbosacral spinal circuitry so that an individual can proactively influence afferent information to enable standing (i.e. by initiating the sitting to standing transition with the assistance of upper limbs) rather than directly inducing a motor task, so precluding or limiting proprioceptive modulation [6]. Hence, the goal was to achieve full body weight-bearing standing with the least amount of assistance while enabling the lumbosacral neural networks to modulate motor pool activity by integrating the postural and weight-bearing related sensory information being projected in real time to the spinal circuitry. These initial results indicate the potential for those even with the diagnosis of clinically sensory and motor complete to regain the ability to stand in their daily life if provided with activity-based rehabilitation combined with individualized epidural stimulation. This capacity not only improves their function but may also combat many of the secondary consequences on health resulting from inactivity.

## Supporting Information

S1 Fig EMG amplitude during sitting and standing. Average (N = 20) peak to peak spinal cord evoked responses amplitude recorded from the four participants during sitting (white bars) and standing (black bars). Stimulation frequency, amplitude and electrode configuration (cathodes in black, anodes in grey, and non-active in white) are reported for each participant. IL: iliopsoas; GL: gluteus maximus; MH: medial hamstring; RF: rectus femoris; VL: vastus lateralis; TA: tibialis anterior; MG: medial gastrocnemius; SOL: soleus. Main effect of sitting versus standing for spinal cord evoked potentials amplitude by Student’s paired t test: *, P ≤ 0.05; £, P ≤ 0.01; **, P ≤ 0.001
(TIF)

S2 Fig EMG and ground reaction forces recorded from participant A53 during standing with stimulation parameters optimal for other individuals. Time course of EMG and ground reaction force recorded from participant A53 during standing with stimulation frequency and electrode configuration that promoted standing with the least amount of assistance for B07 (Panel A), B13 (Panel B) and A45 (Panel C). Stimulation amplitude was adjusted to optimize standing. External assistance to maintain hip and knee extension was needed to stand in all three conditions. IL: iliopsoas; GL: gluteus maximus; MH: medial hamstring; VL: vastus lateralis; TA: tibialis anterior; SOL: soleus.
(TIF)

S3 Fig Effects of anode-cathode electrodes assignment on EMG and standing behaviour. EMG was recorded from participant A45 during standing with stimulation focused on the caudal portion of the lumbosacral spinal cord. Cathodes were either more caudal (Panel A) or more rostral (Panel B) than anodes. Anodes and cathodes were also unbalanced between the lateral columns of the electrode array (Panels C and D). Stimulation frequency was 25 Hz, delivered at an adjusted amplitude to optimize standing. The type of assistance needed for standing, if any, is noted at the top of the figure. Standing attempts reported in Panels B and D were interrupted because of the discomfort caused by the stimulation. Grey dotted lines: change in the type of assistance. Grey shaded area: standing to sitting transition. Stimulation frequency, amplitude and electrode configuration (cathodes in black, anodes in grey, and non-active in white) are reported. GL: gluteus maximus; MH: medial hamstring; VL: vastus lateralis; TA: tibialis anterior; SOL: soleus.
(TIF)

S1 Table Variability of the spinal cord evoked responses during standing at different stimulation frequencies. For all research participants, coefficient of variation was calculated from spinal cord evoked responses (N = 20) selected within a representative portion of continuous (not rhythmic) EMG, recorded during assisted standing with the following stimulation frequencies: 2 Hz, 10 Hz, 20 Hz and 30 Hz. Stimulation amplitude and electrode configuration (cathodes in black, anodes in grey, and non-active in white) are reported below. MH: medial hamstring; VL: vastus lateralis; TA: tibialis anterior; SOL: soleus.
(PDF)

S1 Video Standing with the least amount of assistance performed by the four participants. The stimulation parameters applied for each individual are reported in Fig 1.
(WMV)

S2 Video Sitting to standing transition performed by participant A45. (WMV)

S3 Video Standing promoted by electrode configurations effective and not effective for standing. Standing achieved by participant A45 when the same caudal portion of the lumbosacral spinal cord was stimulated with electrode configurations effective and not effective for standing, respectively. The stimulation parameters applied and the EMG recorded during these two standing attempts are reported in Panels C and D, respectively, in S3 Fig.
(WMV)
