# Supplementary figures and images for: Effects of Lumbosacral Spinal Cord Epidural Stimulation for Standing after Chronic Complete Paralysis in Humans
Source: PLoS One. 2015 Jul 24;10(7):e0133998. doi: 10.1371/journal.pone.0133998 (PMC4514797; doi:10.1371/journal.pone.0133998)

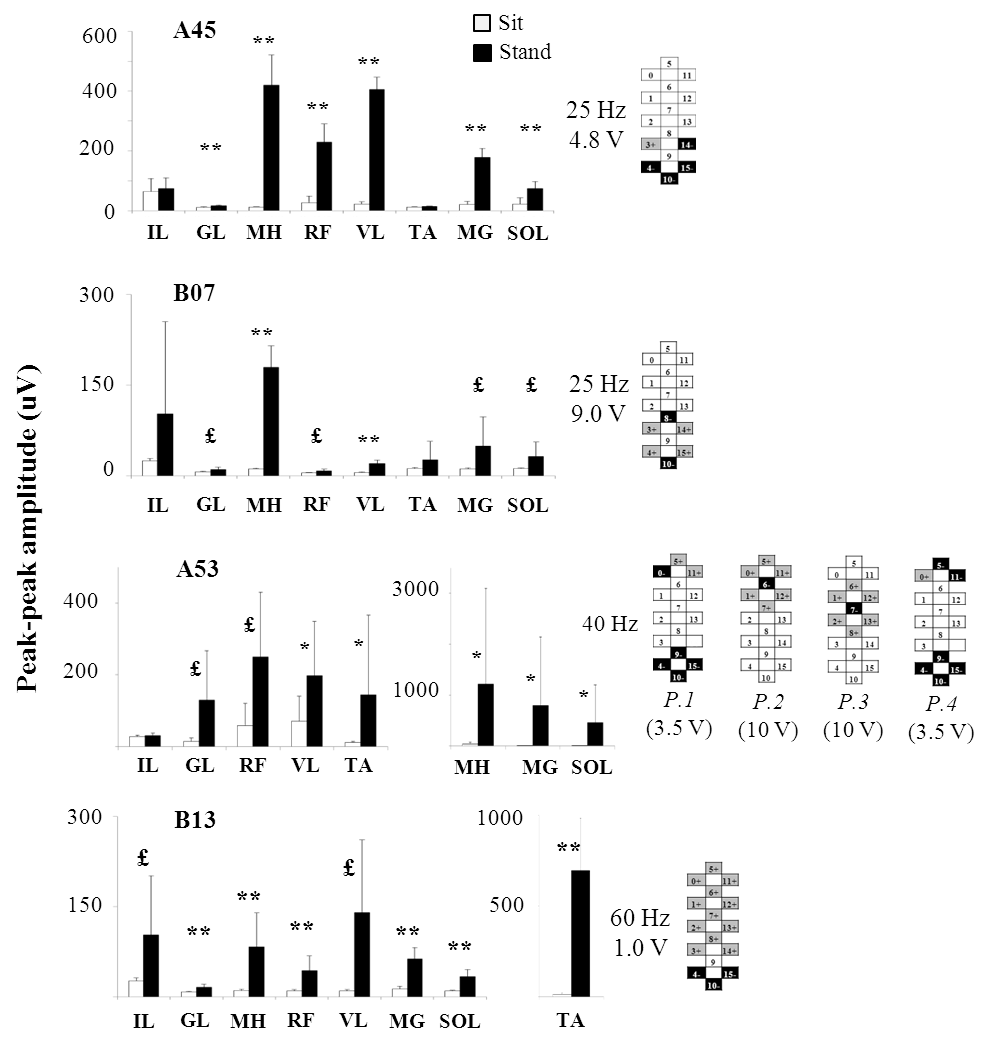

Supplement: S1 Fig — Average (N = 20) peak to peak spinal cord evoked responses amplitude recorded from the four participants during sitting (white bars) and standing (black bars). Stimulation frequency, amplitude and electrode configuration (cathodes in black, anodes in grey, and non-active in white) are reported for each participant. IL: iliopsoas; GL: gluteus maximus; MH: medial hamstring; RF: rectus femoris; VL: vastus lateralis; TA: tibialis anterior; MG: medial gastrocnemius; SOL: soleus. Main effect of sitting versus standing for spinal cord evoked potentials amplitude by Student’s paired t test: *, P ≤ 0.05; £, P ≤ 0.01; **, P ≤ 0.001 (TIF) [file pone.0133998.s001.tif]

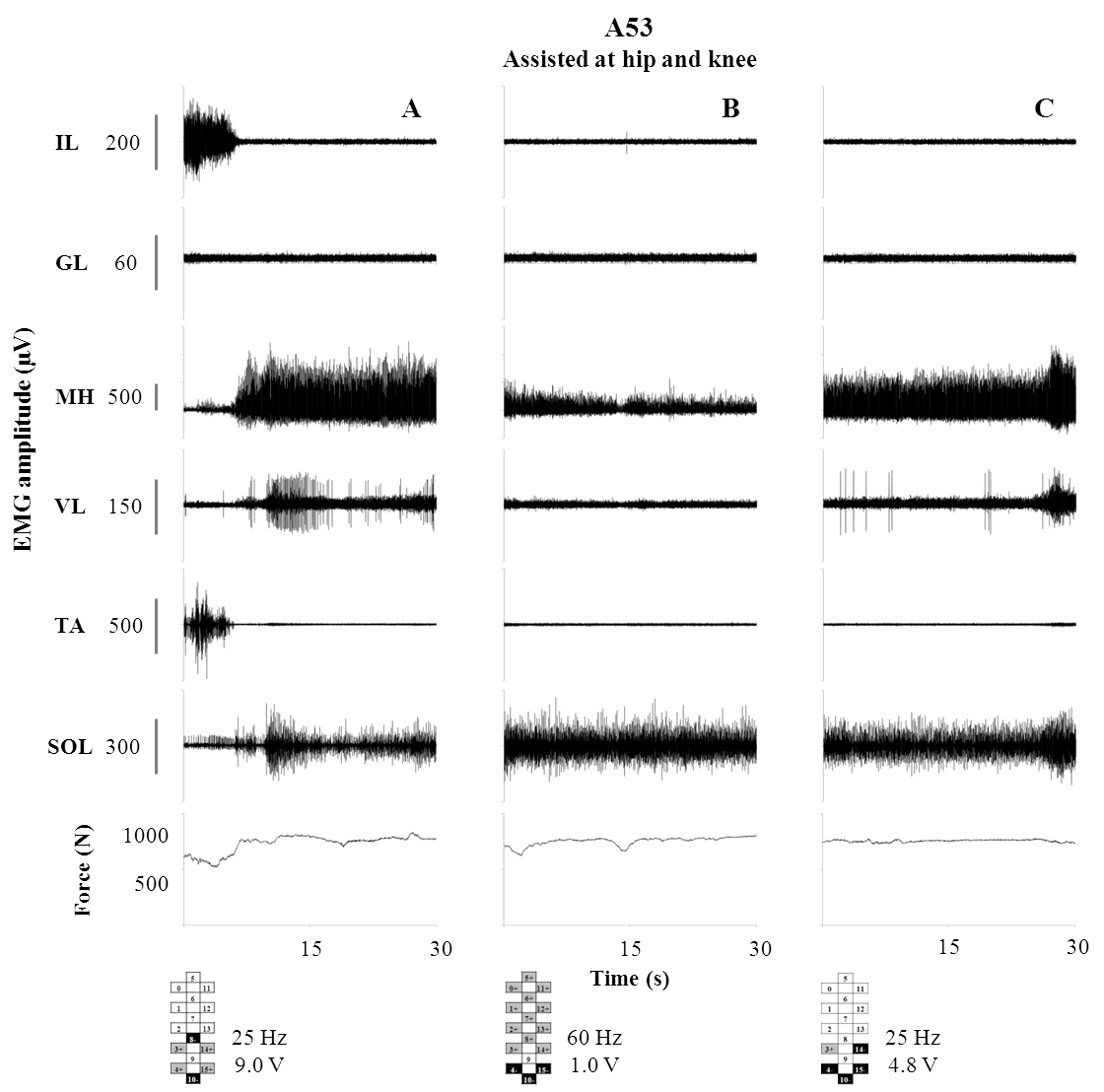

Supplement: S2 Fig — Time course of EMG and ground reaction force recorded from participant A53 during standing with stimulation frequency and electrode configuration that promoted standing with the least amount of assistance for B07 (Panel A), B13 (Panel B) and A45 (Panel C). Stimulation amplitude was adjusted to optimize standing. External assistance to maintain hip and knee extension was needed to stand in all three conditions. IL: iliopsoas; GL: gluteus maximus; MH: medial hamstring; VL: vastus lateralis; TA: tibialis anterior; SOL: soleus. (TIF) [file pone.0133998.s002.tif]

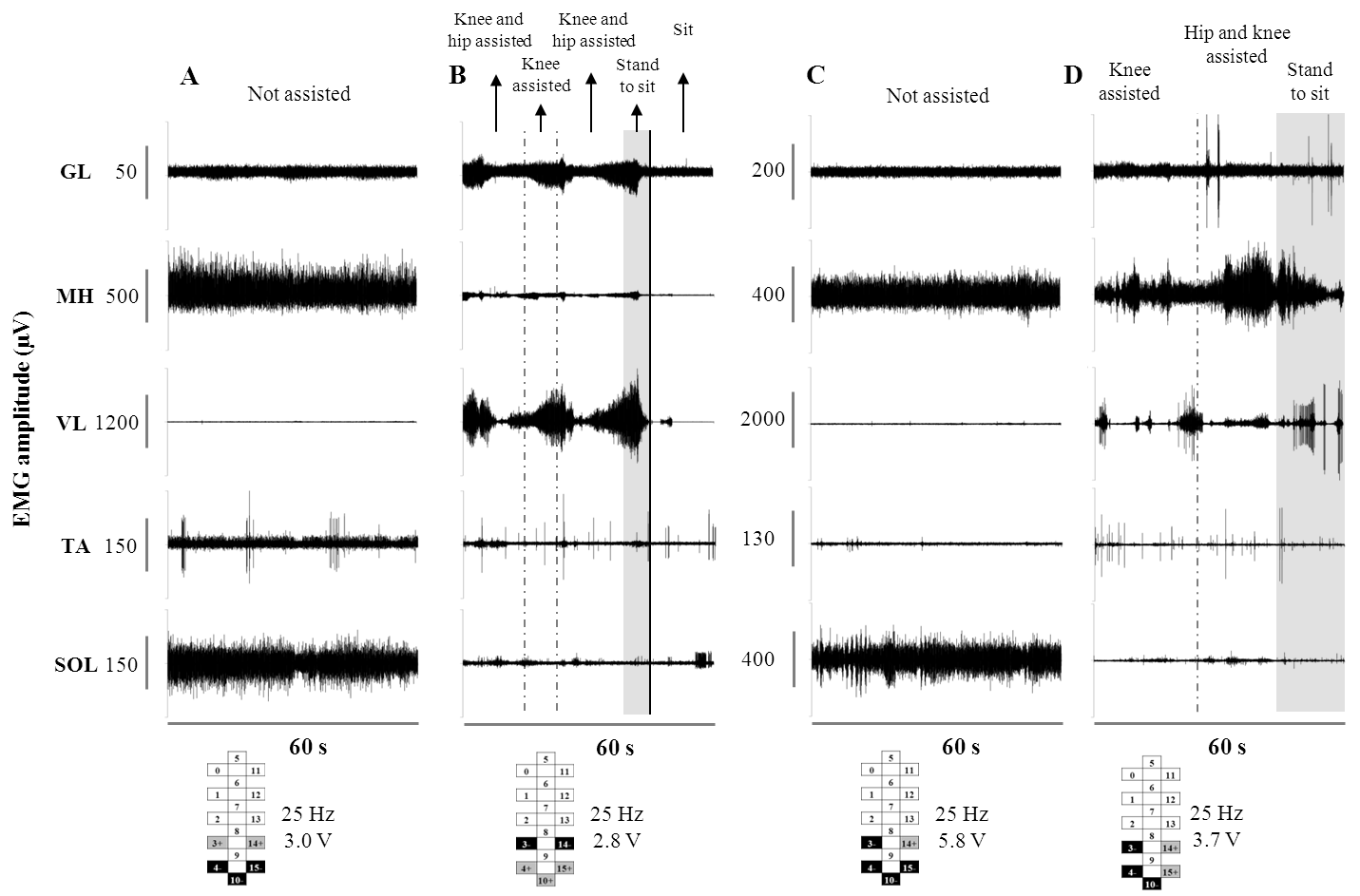

Supplement: S3 Fig — EMG was recorded from participant A45 during standing with stimulation focused on the caudal portion of the lumbosacral spinal cord. Cathodes were either more caudal (Panel A) or more rostral (Panel B) than anodes. Anodes and cathodes were also unbalanced between the lateral columns of the electrode array (Panels C and D). Stimulation frequency was 25 Hz, delivered at an adjusted amplitude to optimize standing. The type of assistance needed for standing, if any, is noted at the top of the figure. Standing attempts reported in Panels B and D were interrupted because of the discomfort caused by the stimulation. Grey dotted lines: change in the type of assistance. Grey shaded area: standing to sitting transition. Stimulation frequency, amplitude and electrode configuration (cathodes in black, anodes in grey, and non-active in white) are reported. GL: gluteus maximus; MH: medial hamstring; VL: vastus lateralis; TA: tibialis anterior; SOL: soleus. (TIF) [file pone.0133998.s003.tif]
