# Supplementary material for: Effects of Lumbosacral Spinal Cord Epidural Stimulation for Standing after Chronic Complete Paralysis in Humans
Source: PLoS One. 2015 Jul 24;10(7):e0133998. doi: 10.1371/journal.pone.0133998 (PMC4514797; doi:10.1371/journal.pone.0133998)

Table S1. Variability of the spinal cord evoked responses during standing at different stimulation frequencies.

For all research participants, coefficient of variation was calculated from spinal cord evoked responses (N = 20) selected within a representative portion of continuous (not rhythmic) EMG, recorded during assisted standing with the following stimulation frequencies: 2 Hz, 10 Hz, 20 Hz and 30 Hz. Stimulation amplitude and electrode configuration (cathodes in black, anodes in grey, and non-active in white) are reported below. MH: medial hamstring; VL: vastus lateralis; TA: tibialis anterior; SOL: soleus.

**Stimulation frequency (Hz)**	**B07**				**A45**				**B13**				**A53**			
	**MH**	**VL**	**TA**	**SOL**	**MH**	**VL**	**TA**	**SOL**	**MH**	**VL**	**TA**	**SOL**	**MH**	**VL**	**TA**	**SOL**
2	0.18	0.18	0.19	0.23	0.22	0.37	0.30	0.18	0.22	0.24	0.33	0.31	0.15	0.22	0.37	0.25
10	0.20	0.44	0.17	0.16	0.10	0.39	0.37	0.31	0.28	0.30	0.25	0.43	0.07	0.41	0.22	0.19
20	0.61	0.61	0.37	0.43	0.23	0.49	0.58	0.61	0.37	0.59	0.60	0.45	0.33	0.58	0.46	0.59
30	0.75	0.69	0.67	0.84	0.50	0.57	0.70	0.59	0.42	0.68	0.59	0.49	0.37	0.84	0.58	0.67

3.0 V

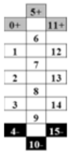

Supplement: S1 Table — For all research participants, coefficient of variation was calculated from spinal cord evoked responses (N = 20) selected within a representative portion of continuous (not rhythmic) EMG, recorded during assisted standing with the following stimulation frequencies: 2 Hz, 10 Hz, 20 Hz and 30 Hz. Stimulation amplitude and electrode configuration (cathodes in black, anodes in grey, and non-active in white) are reported below. MH: medial hamstring; VL: vastus lateralis; TA: tibialis anterior; SOL: soleus. (PDF) [file pone.0133998.s004.pdf]
